# Targeting RARγ Decreases Immunosuppressive Macrophage Polarization and Reduces Tumor Growth

**DOI:** 10.3390/molecules30153099

**Published:** 2025-07-24

**Authors:** Jihyeon Park, Jisun Oh, Sang-Hyun Min, Ji Hoon Yu, Jong-Sup Bae, Hui-Jeon Jeon

**Affiliations:** 1 College of Pharmacy, Kyungpook National University, Daegu 41566, Republic of Korea; bbjh9063@knu.ac.kr; 2 New Drug Development Center, Daegu-Gyeongbuk Medical Innovation Foundation (K-MEDI hub), Daegu 41061, Republic of Korea; joh@kmedihub.re.kr (J.O.); yujihoon@kmedihub.re.kr (J.H.Y.); 3 Department of Innovative Pharmaceutical Sciences, Kyungpook National University, Daegu 41566, Republic of Korea; shmin03@knu.ac.kr; 4 Cell & Matrix Research Institute, Kyungpook National University, Daegu 41566, Republic of Korea; 5 Research Institute of Pharmaceutical Sciences, Kyungpook National University, Daegu 41566, Republic of Korea

**Keywords:** tumor microenvironment, tumor-associated macrophages, M2 polarization, retinoic acid receptor gamma, therapeutic target

## Abstract

Tumor-associated macrophages (TAMs) play a critical role in the tumor microenvironment (TME), interacting with cancer cells and other components to promote tumor growth. Given the influence of TAMs on tumor progression and resistance to therapy, regulating the activity of these macrophages is crucial for improving cancer treatment outcomes. TAMs often exhibit immunosuppressive phenotypes (commonly referred to as M2-like macrophages), which suppress immune responses and contribute to drug resistance. Therefore, inhibiting immunosuppressive polarization offers a promising strategy to impede tumor growth. This study revealed retinoic acid receptor gamma (RARγ), a nuclear receptor, as a key regulator of immunosuppressive polarization in THP-1 macrophages. Indeed, the inhibition of RARγ, either by a small molecule or gene silencing, significantly reduced the expression of immunosuppressive macrophage markers. In a three-dimensional tumor spheroid model, immunosuppressive macrophages enhanced the proliferation of HCT116 colorectal cancer cells, which was significantly hindered by RARγ inhibition. These findings suggest that targeting RARγ reprograms immunosuppressive macrophages and mitigates the tumor-promoting effects of TAMs, highlighting RARγ as a promising therapeutic target for developing novel anti-cancer strategies.

## 1. Introduction

The tumor microenvironment (TME) is a complex and dynamic system composed of tumor cells, blood and lymphatic vessels, extracellular matrix, fibroblasts, and immune cells, all of which interact reciprocally to shape tumor development and progression [1,2,3,4]. Tumor cells actively modify the TME to enhance their survival, proliferation, and metastasis; meanwhile, various stromal and immune components contribute to this process through a network of signaling and structural cues [5,6]. Among these cellular components in the TME, immune cells are the most abundant and undergo metabolic reprogramming that supports tumor growth and immune evasion [7]. Therefore, regulating immune responses within the TME is crucial for achieving effective therapeutic outcomes [8].

Among the immune cell populations in the TME, macrophages are particularly influential due to their plasticity and ability to respond to a broad range of signals within the TME [9]. Depending on contextual cues, macrophages can polarize into either proinflammatory or immunosuppressive phenotypes, thereby exerting dichotomous effects on tumor progression and immune regulation [10,11].

Macrophages play a critical role in both innate and adaptive immunity, characterized by their ability to phagocytose harmful substances, including pathogens, and present antigens [12,13]. Macrophages comprise a heterogeneous population that undergoes activation and functional polarization in response to environmental stimuli, including those derived from the TME [11,14,15]. Meanwhile, naïve M0 macrophages can differentiate into proinflammatory or immunosuppressive phenotypes. Classically activated proinflammatory (M1-like) macrophages exhibit anti-tumorigenic activity by producing proinflammatory cytokines and promoting cytotoxic T-cell responses. In contrast, alternatively activated immunosuppressive (M2-like) macrophages contribute to tumor progression by secreting cytokines, such as IL-6, IL-10, and TGF-β, which facilitate angiogenesis, epithelial–mesenchymal transition, and T-cell modulation, thereby supporting immune evasion, tumor growth, and metastasis [12]. While we acknowledge the functional complexity beyond this binary classification, the M1/M2 terminology is adhered to throughout this manuscript for clarity and consistency.

This phenotypic polarization is orchestrated by complex transcriptional programs regulated by signals within the TME [12,16,17,18]. Among the transcriptional regulators involved, nuclear receptors, which are ligand-activated transcription factors traditionally associated with development, metabolism, and homeostasis, have recently emerged as potential modulators of macrophage polarization [12,17]. However, the specific roles of nuclear receptors in the acquisition and maintenance of the tumor-supportive features in M2 macrophages remain poorly understood.

Tumor-associated macrophages (TAMs) are the most abundant immune cells in the TME, accounting for more than 50% of the total tumor mass [11,19]. Moreover, TAMs exhibit diverse functions across various cancer types yet predominantly display a protumorigenic M2-like phenotype [15,20,21,22]. The density and polarization state of TAMs, particularly those with an M2-like phenotype, are strongly correlated with poor clinical outcomes in various malignancies [3,23]. Thus, targeting or modulating TAM polarization represents a promising strategy for cancer therapy [16,24,25]. This study aimed to investigate methods to counteract the tumor-promoting effects of M2-like macrophages, with a particular focus on nuclear receptors as transcriptional regulators of immunosuppressive polarization within the TME.

## 2. Results

### 2.1. IL-4 and IL-13 Treatments Induce M2 Polarization of M0 Macrophages

**Figure 1:**
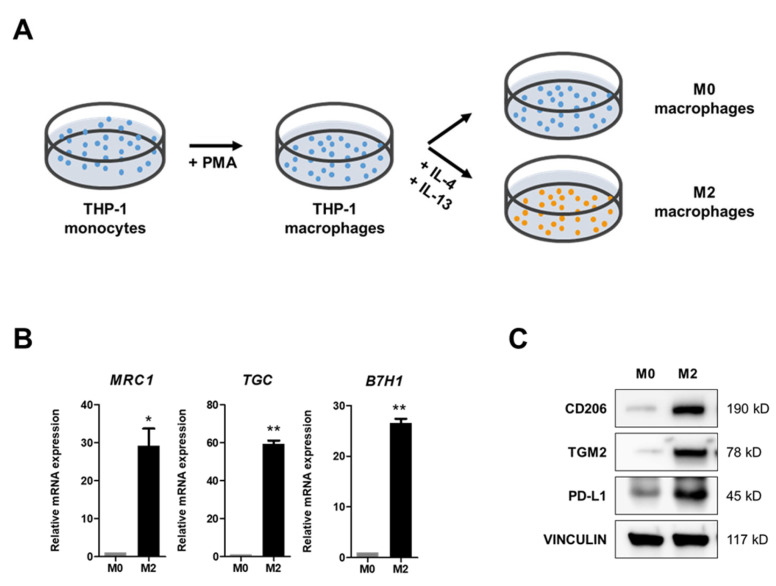
M2 polarization of THP-1 macrophages by IL-4 and IL-13. (**A**) Experimental scheme for THP-1 cell culture. THP-1 cells were treated with 50 nM PMA for 48 h, followed by treatment with 20 ng/mL of human recombinant IL-4 and IL-13 for an additional 48 h to induce M2 polarization. (**B**,**C**) Expression of M2 polarization markers at the mRNA (**B**) and protein (**C**) levels, including *MRC1*, *TGC*, and *B7H1*, which encode mannose receptor type C (CD206), transglutaminase 2 (TGM2, C polypeptide), and programmed cell death 1 ligand 1 (PD-L1), respectively. Statistical significance was expressed by asterisks in comparison with the control (M0): *, *p* < 0.05; **, *p* < 0.01.

To investigate the role of M2 macrophages in tumor growth, we differentiated THP-1 cells with PMA for 48 h and subsequently induced M2 polarization through additional treatments with human recombinant IL-4 and IL-13 for 48 h (Figure 1A). The THP-1 cells treated with IL-4 and IL-13 displayed an elongated M2 macrophage-like shape, while the cells treated with PMA alone retained a round morphology of M0 macrophages (Supplementary Figure S1). The M2 polarization of THP-1 cells was evaluated through the expression of M2 markers, including *MRC1*, *TGC*, and *B7H1* encoding mannose receptor type C (CD206), transglutaminase 2 (TGM2, C polypeptide), and programmed cell death 1 ligand 1 (PD-L1), respectively (Figure 1B,C). We found that cells treated with IL-4 and IL-13 expressed M2 marker expression at significantly higher levels compared to controls, both in mRNA and protein levels, suggesting the successful induction of M2 polarization (i.e., differentiation into an immunosuppressive/protumoral phenotype) through treatment of M0 THP-1 cells in our culture conditions with IL-4 and IL-13.

### 2.2. A Heterodimer of RARγ and RXRγ Mediates M2 Polarization

**Figure 2:**
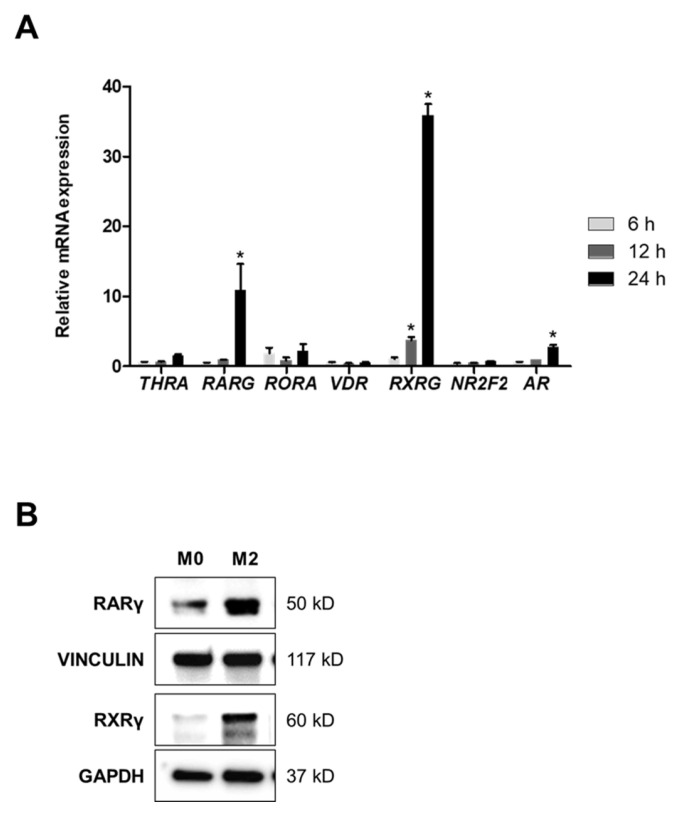
Upregulation of RARγ and RXRγ in M2 THP-1 macrophages. (**A**) PMA-treated THP-1 cells were treated with IL-4 and IL-13 for 6 h, 12 h, and 24 h, followed by analysis using qPCR. (**B**) M0 and M2 THP-1 cells were harvested and analyzed for the expression of RARγ and RXRγ proteins. Statistical significance was expressed by asterisks (*, *p* < 0.05) in comparison with values at 6 h.

Nuclear receptors, which act as transcription factors, are influenced by external factors due to their ligand-dependent activation. Thus, we hypothesized that nuclear receptors may play a role in the polarization of macrophages. We selected 42 nuclear receptors with characterized genetic information and assessed changes in their expression levels using qPCR in M2 THP-1 cells compared with M0 cells. Several receptors showed a significant increase in mRNA expression in M2 cells after treatment with IL-4 and IL-13 for 48 h (Supplementary Table S1). Notably, the gene *RXRG*, which encodes retinoid X receptor gamma (RXRγ), was remarkably upregulated under polarization-inducing conditions. Interestingly, *RARG*, which encodes retinoic acid receptor gamma (RARγ)—an isotype of RARs that works in the form of a heterodimer with RXRs involving RXRγ in response to retinoic acids (RAs) [26] abundantly found in the TME [27]—was also highly expressed alongside the increased expression of RXRG (Figure 2A). RXRG demonstrated a 3.7-fold increase at 12 h and a 35.8-fold increase at 24 h, while RARG showed an approximately 10-fold increase at 24 h. Furthermore, the protein levels of RARγ and RXRγ were significantly elevated in M2 THP-1 cells compared with M0 cells (Figure 2B). These findings suggest the RARγ–RXRγ heterodimer is likely involved in M2 macrophage polarization.

### 2.3. Inhibition of RARγ Suppresses M2 Polarization

**Figure 3:**
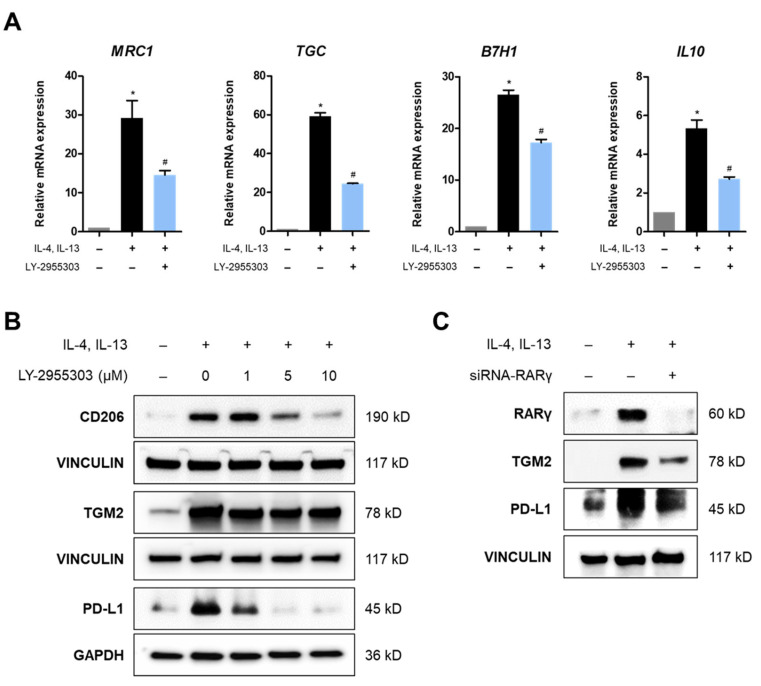
Suppression of M2 polarization through RARγ inhibition. (**A**,**B**) PMA-treated THP-1 cells were cultured in the M2 polarization-inducing medium containing IL-4 and IL-13, with or without the RARγ inhibitor LY-2955303. Cells were harvested and analyzed for M2 marker expression at the mRNA level for *MRC1*, *TGC*, *B7H1*, and *IL-10* (**B**), and the protein level for CD206, TGM2, and PD-L1. (**C**) PMA-treated THP-1 cells were transfected with siRNA targeting *RARγ* and analyzed for protein expression of RARγ and M2 markers, including TGM2 and PDL-1. Statistical significance was expressed by asterisks (*, *p* < 0.05) compared with the untreated control, and by hashtags (#, *p* < 0.05) compared with the IL-4/IL-13-treated group.

Based on the observation that M2 THP-1 cells significantly expressed RARγ and RXRγ, we examined whether inhibiting the RARγ–RXRγ heterodimer affects M2 polarization. Since specific small-molecule inhibitors against RXRγ are unavailable, we utilized the RARγ-selective inhibitor, LY-2955303 (Supplementary Figure S2A). LY-2955303 was added at a concentration of 10 μM, which was non-cytotoxic to THP-1 cells (Supple. Figure S2B), under M2 polarization-inducing conditions with IL-4 and IL-13. The qPCR results showed that the relative mRNA expression levels of M2 macrophage markers, including *MRC1*, *TGC*, *B7H1*, and *IL10*, were significantly increased in the polarization-inducing conditions; however, treatment with the RARγ inhibitor reduced their expression levels despite the presence of IL-4 and IL-13 (Figure 3A). In addition, the protein levels of TGM2, CD206, and PD-L1 were markedly increased with M2 polarization induction compared with the control but decreased in LY-2955303-treated cells (Figure 3B). Furthermore, *RARG* knockdown by siRNA during M2 polarization resulted in a significant reduction in TGM2 and PD-L1 protein expression (Figure 3C). These findings demonstrate that disrupting RARγ either through chemical inhibition or gene silencing suppresses the M2 polarization of THP-1 cells, indicating the acquisition of an immunosuppressive phenotype. Moreover, these findings suggest that RARγ, as a heterodimeric partner of RXRγ, may serve as a promising target for inhibiting the polarization of M2 macrophages in the TME.

### 2.4. RARγ Inhibitor Impedes Macrophage-Associated Tumor Growth in Spheroid Culture

**Figure 4:**
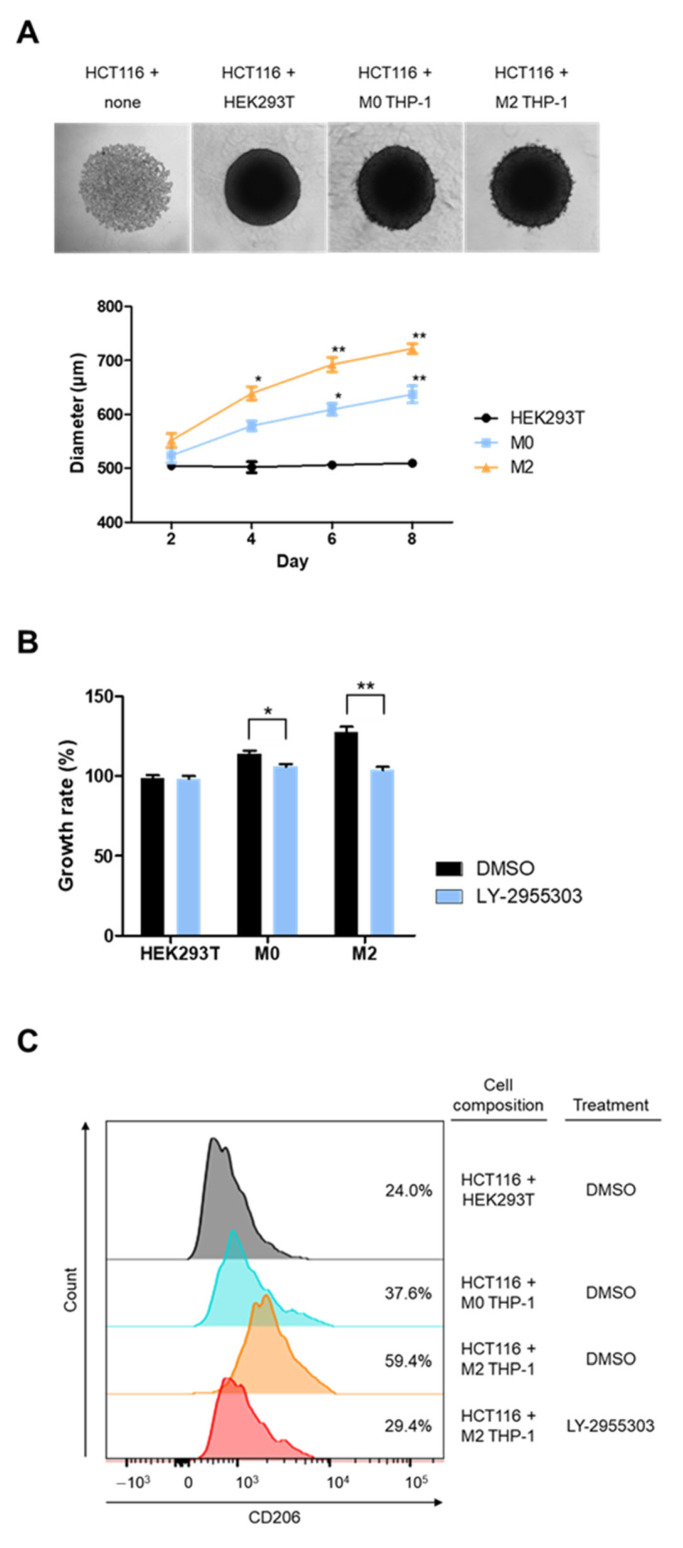
M2 macrophage-mediated tumor growth in spheroids. (**A**) A 3D spheroid model was generated by co-culturing M0 or M2 THP-1 cells with HCT116 cells at a 1:5 ratio, and the spheroids were monitored for up to 8 days. HEK293T cells were used as a control, showing minimal influence on HCT116 cells. Spheroids co-cultured with M2 THP-1 cells showed a significant increase in size compared with M0 THP-1 spheroids, starting from day 6. (**B**) Treatment with the RARγ inhibitor, LY-2955303, significantly reduced spheroid size in the THP-1 co-cultures by day 6. (**C**) FACS analysis revealed a significant reduction in the number of CD206-positive M2 THP-1 cells in LY-2955303-treated spheroids. Statistical significance was expressed by asterisks in comparison with the control condition: *, *p* < 0.05; **, *p* < 0.01.

To examine the effectiveness of RARγ inhibition in suppressing tumor growth, THP-1 cells before and after M2 polarization were co-cultured with HCT116 cells in a three-dimensional (3D) spheroid formation (Figure 4A). Spheroids were generated either by mixing THP-1 and HCT116 cells at a 1:5 ratio or by culturing HCT116 cells alone and were maintained for up to 8 days. The average sizes of the spheroids in the culture increased over time in the presence of THP-1 cells, but not in spheroids composed of HCT116 cells alone (Figure 4; Supplementary Figure S3). In addition, spheroids co-cultured with M2 THP-1 cells exhibited a significantly larger size compared with those with M0 THP-1 cells starting from day 6. Importantly, the increase in size in the presence of THP-1 cells was hindered by treatment with the RARγ inhibitor, LY-2955303 (Figure 4B). Indeed, LY-2955303 treatment reduced the count of CD206-positive cells in spheroids cultured with M2 THP-1 cells (Figure 4C). These findings suggest that RARγ inhibition disrupts the M2 polarization of THP-1 cells in spheroids formed with HCT116 colorectal cancer cells, thereby impeding tumor growth.

## 3. Discussion

This study demonstrates the critical role of the nuclear receptor RARγ in regulating M2 macrophage polarization and its impact on tumor growth in the TME. Our findings indicate the potential therapeutic significance of targeting M2 macrophages, which are known to promote tumor progression, suggesting that RARγ could serve as a promising target for cancer treatment.

TAMs constitute the most abundant immune cell population infiltrating the TME [8,19,23,28]. Macrophages can be classified into two subtypes based on their polarization states: the classically activated M1 subtype and the alternatively activated M2 subtype. M2 macrophages are well recognized for their immunosuppressive, pro-angiogenic, and extracellular matrix remodeling functions, which contribute to enhanced tumor survival and the suppression of anti-tumor immune responses. Under our culture conditions, PMA-treated THP-1 cells were successfully polarized to the M2 phenotype through treatment with IL-4 and IL-13, as demonstrated by the significant upregulation of M2 markers including CD206, TGM2, and PD-L1.

Importantly, we observed that RARγ, a nuclear receptor involved in cellular differentiation and proliferation in response to RAs [29,30], was highly expressed in THP-1-derived M2 macrophages. Consistent with the fact that RARs exert their biologic effects through heterodimerization with RXRs [31], our results demonstrated a significant, coordinated upregulation of RARγ and RXRγ in M2-polarized cells. This suggests that the RARγ-RXRγ heterodimer plays a pivotal role in regulating macrophage polarization, possibly through the RA signaling pathway, which is enriched in the TME.

Pharmacological inhibition of RARγ using LY-2955303 restricted M2 polarization. qPCR and Western blot analyses demonstrated that LY-2955303 treatment significantly downregulated the mRNA and protein levels of M2 markers in THP-1 cells, indicating that RARγ is essential for M2 polarization. RARG knockdown by siRNA also reduced M2 marker expression, confirming the involvement of RARγ in this process. These results provide functional evidence that RARγ is not only associated with but also mechanistically essential for maintaining M2-specific gene expression. Furthermore, LY-2955303 treatment impeded tumor growth in the spheroid model. In this system, HCT116 colorectal cancer cells were co-cultured with THP-1 macrophages, where M2-polarized macrophages significantly enhanced tumor spheroid growth, consistent with their known role in promoting tumor cell proliferation, survival, and invasion [9,16,32]. However, RARγ inhibition by LY-2955303 significantly reduced spheroid size and decreased the number of CD206-positive macrophages within the spheroids.

In addition to its direct role in regulating macrophage polarization, RARγ inhibition may influence the immunological composition and functional dynamics of the TME. Since macrophages regulate the activity of other immune cells through cytokine secretion and antigen presentation, suppressing M2 polarization could reduce local immunosuppression and enhance anti-tumor immune responses [9,12,33]. Although this study primarily utilized THP-1-derived macrophages, the observed upregulation in RARγ and RXRγ during M2 polarization suggests that this regulatory mechanism may be conserved in primary human macrophages and tumor-associated macrophages in vivo. Moreover, nuclear receptors are known to interface with epigenetic regulators and chromatin-modifying complexes [34,35], implying that RARγ inhibition could drive the stable reprogramming of macrophage identity, potentially leading to long-lasting changes in immune cell behavior within the TME.

Moreover, integrating RARγ-targeted approaches with existing immunotherapeutic strategies may offer synergistic benefits in cancer treatment. For instance, immune checkpoint inhibitors, such as anti-PD-1 or anti-CTLA-4 antibodies, have shown limited efficacy in tumors with a highly immunosuppressive TME dominated by M2-like TAMs [16,36,37]. By reprogramming macrophages toward a less suppressive or proinflammatory phenotype through RARγ inhibition, the responsiveness to checkpoint blockade may be significantly enhanced [38,39,40]. In addition, since RARγ is a nuclear receptor that small molecules can modulate, this strategy holds translational potential for combination therapies with conventional chemotherapy or radiotherapy [30,41], which are known to induce immunogenic cell death. Therefore, future studies should investigate the timing, dosing, and sequencing of RARγ inhibition in combination regimens to determine the most effective clinical application. While the present study demonstrates functional interactions between macrophages and tumor cells, this study did not include dual immunofluorescence staining to directly visualize their spatial relationships within the spheroid microenvironment. Thus, this limitation will be addressed in future work through imaging-based approaches, which will provide more direct evidence of cellular crosstalk. After confirming macrophage differentiation, THP-1-derived macrophages were co-cultured with HCT116 tumor cells at a 2:1 ratio in spheroid conditions for 7 days.

In conclusion, this study demonstrates that RARγ plays a crucial role in the polarization of M2 macrophages and tumor growth. The inhibition of RARγ disrupted M2 polarization and diminished the tumor-supportive functions of macrophages in the TME, thereby reducing tumor growth in a 3D spheroid model. These findings underscore that RARγ may serve as a promising therapeutic target for modulating macrophage activity in cancer, offering potential for the development of novel anti-cancer therapies that aim to alter the immune responses within the TME. Nonetheless, further studies are warranted to explore the broader applicability of RARγ inhibitors in other cancer models and to investigate their potential synergistic effects when combined with existing cancer immunotherapies. Future research should examine the pharmacodynamics and safety profile of RARγ inhibition in vivo, optimizing the dosage and delivery strategies to maximize therapeutic benefit while minimizing the off-target effects.

## 4. Materials and Methods

### 4.1. Cell Culture

All cell lines used in this study were obtained from ATCC (Manassas, VA, USA). All culture media and supplements were purchased from Cytiva (Marlborough, MA, USA) unless otherwise stated. The human monocyte cell line THP-1 was maintained in RPMI 1640 medium supplemented with 10% fetal bovine serum (FBS) and 1% penicillin/streptomycin. The cells were grown in suspension and subcultured by diluting single-cell suspensions in maintenance medium every 2–3 days. For differentiation of THP-1 cells into M0 macrophages, cells were seeded in a 100 mm culture dish (Corning, Corning, NY, USA) at a density of 3.5 × 10^6^ and treated with 50 nM phorbol 12-myristate 13-acetate (PMA) (R&D Systems, Minneapolis, MN, USA) for 48 h. To induce M2 polarization, the differentiated cells were additionally treated with 20 ng/mL of human recombinant IL-4 and IL-13 for 48 h [42,43]. Adherent THP-1 cells were detached using Accutase (STEMCELL Technologies, Vancouver, BC, Canada) or cell scrapers (Gibco/Thermo Fisher Scientific, Waltham, MA, USA). Cells were imaged using a Leica microscope with Leica LAS EZ software (version 2.1.0, Leica Microsystems, Wetzlar, Germany).

### 4.2. Spheroidal Co-Culture of THP-1 and HCT116 Cells

HCT116 human colorectal cancer cells were cultured in RPMI 1640 medium supplemented with 10% FBS and 1% penicillin/streptomycin and subcultured using trypsin–EDTA at approximately 80% confluency. For spheroidal cultures, HCT116 cells were seeded in ultra-low attachment multiple-well plates (round-bottom, clear, 96 wells; Corning) at a density of 5 × 10^3^ cells per well, along with M0 or M2 THP-1 cells at a density of 1 × 10^3^ cells per well. Cells were incubated in 100 μL of RPMI 1640-based maintenance medium for up to 8 days, with media refreshed every other day. Spheroids were imaged and their diameters measured using a Cytation 5 reader (BioTek Instruments, Inc., Winooski, VT, USA) starting two days after seeding. LY-2955303 (Sigma-Aldrich, St. Louis, MO, USA) was added from day 2.

### 4.3. Cell Viability Assay

Cells were seeded in white 96-well plates (Corning) at the designated density per well. Cell viability was assessed using the CellTiter Glo^®^ Luminescent Cell Viability assay (Promega Corp., Madison, WI, USA). The relative luminescence intensity, corresponding to the number of viable cells, was measured using a microplate reader (TECAN, Männedorf, Switzerland).

### 4.4. Real-Time Quantitative PCR (qPCR) Analysis

Total RNA was extracted from the harvested THP-1 cells using TRIzol reagent (Thermo Fisher Scientific), followed by treatment with DNase I (Thermo Fisher Scientific) to remove genomic DNA contamination. A total of 5 μg of the extracted RNA was then reverse-transcribed into cDNA using the GoScript^TM^ cDNA synthesis system (Promega), which served as a template for qPCR. Next, qPCR was performed using PowerUp^TM^ SYBR^TM^ Green Master Mix (Applied Biosystems, Waltham, MA, USA) on a StepOnePlus^TM^ Real-Time PCR System (Applied Biosystems). The mRNA levels of the genes of interest were quantified relative to the housekeeping gene, *36B4*, and relative expression was calculated using the 2^−ΔΔCt^ method. Primers were designed using Primer-BLAST (NCBI, Bethesda, MD, USA) with parameters set to span exon–exon junctions and avoid secondary structures. The primer sequences are listed in Table 1. All primers were validated for specificity and amplification efficiency through melt curve analyses and serial dilution before use in experiments.

### 4.5. Western Blot Analysis

Cells were harvested and lysed in RIPA buffer (Thermo Fisher Scientific) supplemented with a protease and phosphatase inhibitor cocktail (Thermo Fisher Scientific) and phenylmethylsulfonyl fluoride (Sigma-Aldrich). Following centrifugation, the proteins in the cell lysates were isolated and their quantities were measured using a bicinchoninic acid (BCA) protein assay kit (Thermo Fisher Scientific) according to the manufacturer’s instructions. The protein samples (20 µg protein per lane) were then electrophoretically separated using sodium dodecyl sulfate–polyacrylamide gel electrophoresis (SDS-PAGE) (Bio-Rad, Hercules, CA, USA) and transferred onto a polyvinylidene difluoride (PVDF) membrane (Sigma-Aldrich). After blocking with a 5% skim milk solution, the membrane was incubated with primary antibodies at 4 °C overnight, followed by incubation with secondary antibodies at room temperature for 1 h. The antibodies used in this study are detailed in Table 2. Antibody binding was visualized using ECL Western Blotting Substrate (Thermo Fisher Scientific, USA) and detected by LuminoGraph III Lite (ATTO, Tokyo, Japan). The results were quantified and analyzed by ImageJ software (version 1.53; National Institutes of Health, Bethesda, MD, USA).

### 4.6. siRNA Transfection

THP-1 cells were plated in a T75 flask (Corning) at a concentration of 1.5 × 10^7^ and differentiated using 50 nM of PMA for 48 h. The adherent cells were detached using Accutase, harvested, and replated in 6-well plates at a density of 1.5 × 10^6^ cells per well in Opti-MEM medium (Gibco/Thermo Fisher Scientific). M0 THP-1 cells were transfected with siRNA targeting *RARG* or non-specific control siRNA (Bioneer, Daejeon, South Korea) using Lipofectamine RNAiMax Transfection reagent (Thermo Fisher Scientific) according to the manufacturer’s instructions. The transfected M0 THP-1 cells were subsequently treated with 20 ng/mL of IL-4 and IL-13 to induce M2 polarization. M0 and M2 THP-1 cells were harvested and subsequently subjected to Western blot analysis.

### 4.7. Fluorescence-Activated Cell Sorting (FACS)

The harvested cells were resuspended in FACS buffer (phosphate-buffered saline containing 2% FBS and 0.1% sodium azide) and incubated with the primary anti-CD206 antibody, followed by incubation with a FITC-conjugated secondary antibody. The cells were then washed with FACS buffer to remove excess antibody. CD206-positive cells were sorted using a BD FACSAria III (BD Biosciences, Franklin Lakes, NJ, USA), with optimized parameters.

### 4.8. Statistical Analysis

Values are expressed as the mean ± standard deviation (SD). The normality of the data was assessed using the Shapiro–Wilk test, and homogeneity of variance was evaluated using Levene’s test. The two groups were assessed using an unpaired Student’s *t*-test. Statistical analyses were conducted using GraphPad Prism 8 software (GraphPad Software Inc., La Jolla, CA, USA), and a *p*-value of <0.05 was considered statistically significant.

## Figures and Tables

**Table 1:** List of primer sequences for qPCR.

Gene	Primer Sequences (5′ → 3′)	
*THRA*	F	GAAAGCGAAAAAGAAAGAACGG
	R	AGGAATAGGTGGGATGGAGG
*RARA*	F	ACGAGAGTGTAGAAGTGGG
	R	TTGTAGATGCGGGGTAGAG
*RARB*	F	ACCATCGCAGACCAAATTAC
	R	TCATCCATTTCCAAAGGCAG
*RARG*	F	GGTCTACAAGCCATGCTTC
	R	ACCCTTCTTCCTTCACCTC
*PPARA*	F	CGCAAACTTGGACCTGAAC
	R	AGCAAATGATAGCAGCCAC
*PPARD*	F	GGGAAGAGGAGGAGAAAGAG
	R	CATGAACACCGTAGTGGAAG
*PPARG*	F	TTCAAACACATCACCCCCC
	R	GCTCTTTAGAAACTCCCTTGTC
*NR1D1*	F	TCCCCTTCTTCCTCATCTTC
	R	GTCCCCACACACTTTACAC
*NR1D2*	F	ATGGAGGTGAATGCAGGAG
	R	TGGAGAAGATGGAACAGAAGAG
*RORA*	F	ACATACAGCCTTCCCCAGAC
	R	AATTGCCACATCACCTCCC
*RORB*	F	ACAATGCTTCTTATTCCTGCC
	R	GACATCCTCCCAAACTTCAC
*NR1F3*	F	AAAGCAGGAGCAATGGAAG
	R	GGGAGTGGGAGAAGTCAAAG
*NR1H2*	F	CTTCCACTACAACGTGCTC
	R	ACTGTGACTGTGACTCCTG
*NR1H3*	F	TGCCTGACATTCCTCCTGAC
	R	GCCCCTTTTTCCGCTTTTG
*NR1H4*	F	AACATTCCCATTTACCTACCAC
	R	TGCTACCTCAGTTTCTCCC
*VDR*	F	ATCACCAAGGACAACCGAC
	R	TCCCTCCACCATCATTCAC
*NR1I2*	F	GCCAAAGTCATCTCCTACTTC
	R	ATCAGCACATACTCCTCCTC
*HNF4A*	F	AAGCCATCATCTTCTTTGACC
	R	ATCTGCTCGATCATCTGCC
*HNF4G*	F	CAACATCCCCTCCATTAACAC
	R	TAGCCCATTCCACCAAGAC
*RXRA*	F	CTTCCTTCACCAAGCACATC
	R	ACTCCACCTCATTCTCGTTC
*RXRB*	F	GCTTCTTCAAACGCACCATC
	R	ATCCCCATCCTTGTCCTTTC
*RXRG*	F	GCAAGAAGAAAGACAGAGGAG
	R	TGCCCGAAGCAAAATGAC
*NR2C1*	F	GATAATTCTCCAGACCAAGGAC
	R	GCTTCATTCCAAACGCAATAC
*NR2C2*	F	TGGCAGATGGGATAGACAC
	R	TGAGAGGAAAAGCAGACGG
*NR2E1*	F	AGGAATGGGGAAAAAGAGGG
	R	ATGGACAGACAGATGGAGAG
*NR2E3*	F	CCTCCTCTCCATACTCCTCTTC
	R	AGTTCACTCCACGCCTCTTC
*NR2F1*	F	CCGCAGGAACTTAACTTACAC
	R	GCATTCTTCCTCGCTGAAC
*NR2F2*	F	TCCAAGAGCAAGTGGAGAAG
	R	AAGGGAGGCGAAGCAAAAG
*ESR1*	F	ATCTGCCAAGGAGACTCGCTAC
	R	TCCCACCTTTCATCATTCCCAC
*ESR2*	F	ATGCTCACTTCTGCGCTGTC
	R	CACACTTCACCATTCCCACTTC
*ESRRA*	F	AAGACAGCAGCCCCAGTGAATG
	R	AGACAGCGACAGCGATGAGAAG
*ESRRB*	F	AAGCACATCCCAGGCTTCTC
	R	CACAAACTCCTCCTTCTCCAC
*ESRRG*	F	TTCAGCCAGCCAAAAAGCC
	R	TCGCCCATCCAATGATAACC
*NR3C1*	F	TTACCACAACTCACCCCTAC
	R	TGCCTTTGCCCATTTCAC
*AR*	F	TGCCCATTGACTATTACTTTCC
	R	TCCCTGCTTCATAACATTTCC
*NR4A1*	F	TTCAAAACCCAAGCAGCC
	R	TACATCCCCAGCATCTTCC
*NR4A2*	F	TGACACCCAGCATATCCAG
	R	CAATCCATTCCCCAAAGCC
*NR5A1*	F	ACAGCAGAAGAAGGCACAG
	R	GAAAGGCAGGGTAGAGGTAG
*NR5A2*	F	ACCAGAACTGCCAAATTGAC
	R	CCCAAACTTATTCCTTCCTCC
*NR6A1*	F	TATTTGCCCTGCTTTGCC
	R	ATCACCTCCATCCCTTCATC
*NR0B1*	F	TGCAGAAGATCCTCACCAC
	R	CAGCATTTGGAAAGAAAGCAC
*NR0B2*	F	GCCCAGCATACTCAAGAAG
	R	TCCAGACAGCATTGAAGCC
*36B4*	F	CAGCAAGTGGGAAGGTGTAATCC
	R	CCCATTCTATCATCAACGGGTACAA

**Table 2:** List of primary antibodies for Western blot analysis.

Antibody	Source	Catalog #	Dilution	Application
TGM2	Cell Signaling Technology (Danvers, MA, USA)	#3557	1:2000	WB
CD206	Cell Signaling Technology	#91992	1:2000	WB
CD206	Santa Cruz Biotechnology (Dallas, TX, USA)	#sc-376232	1:100	FACS
PD-L1	Abcam (Waltham, MA, USA)	#ab210931	1:2000	WB
RAR-γ	Cell Signaling Technology	#8965	1:2000	WB
RXR-γ	Santa Cruz Biotechnology	#sc-365252	1:2000	WB
Vinculin	Santa Cruz Biotechnology	#sc-73614	1:2000	WB
GAPDH	Santa Cruz Biotechnology	#sc-47724	1:2000	WB

## Data Availability

The authors declare that all data supporting the findings of this study are available in the article and can be provided by the corresponding author upon reasonable request.

## Supplementary Materials

The following supporting information can be downloaded at: https://www.mdpi.com/article/10.3390/molecules30153099/s1, Table S1: relative mRNA expression of 42 genes in M2 compared to M0 THP-1 cells. Figure S1: representative images of THP-1 cells. (A) M0 THP-1 cells were treated with PMA for 48 h. (B) M2 THP-1 cells cultured in polarization-inducing medium containing IL-4 and IL-13; Figure S2: information on LY-2955303. (A) Chemical structure. (B,C) Cell viability of THP-1 (B) and HCT116 cells (C) treated with various concentrations of the chemical, demonstrating no cytotoxicity observed; Figure S3: three-dimensional spheroids of HCT116 cells were cultured and monitored for changes in size for 8 days. Spheroids composed of HCT116 cells alone showed no significant increase in size during the experimental period, and their sizes were also unaffected by LY-2955303 treatment.
