# Supplementary material for: Targeting RARγ Decreases Immunosuppressive Macrophage Polarization and Reduces Tumor Growth
Source: Molecules. 2025 Jul 24;30(15):3099. doi: 10.3390/molecules30153099 (PMC12348448; doi:10.3390/molecules30153099)
Supplement: Supplementary file 1 [file molecules-30-03099-s001.zip › molecules-3753308-supplementary.pdf]

Suppl. Figure S1

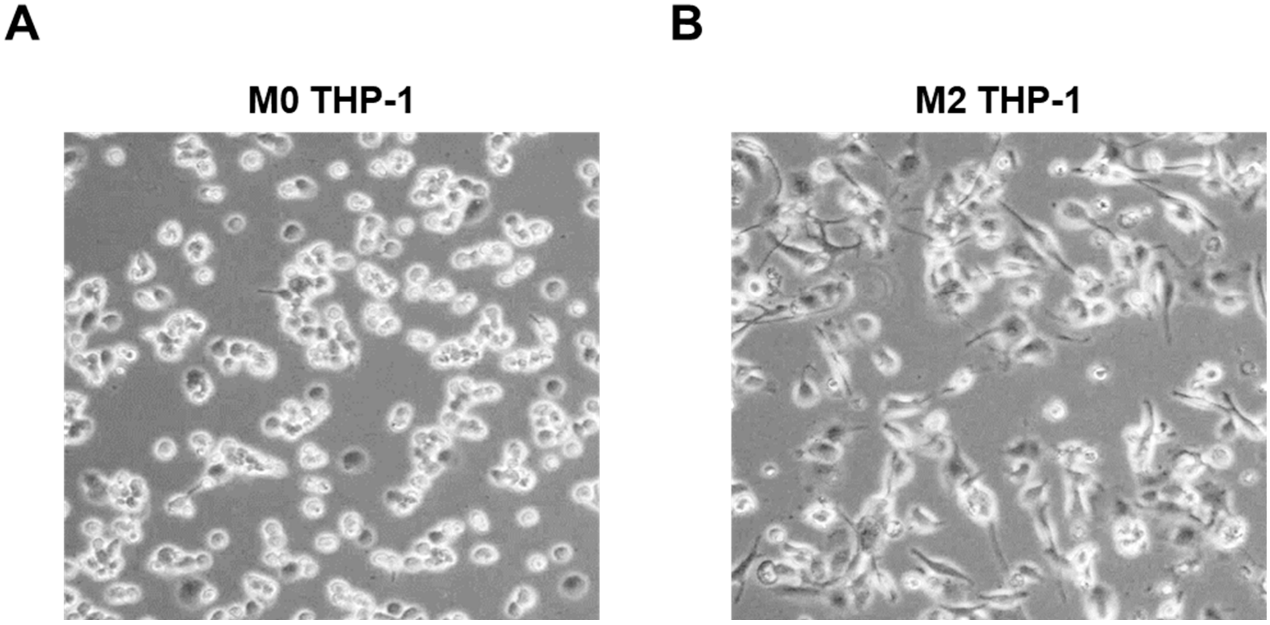

Suppl. Figure S2

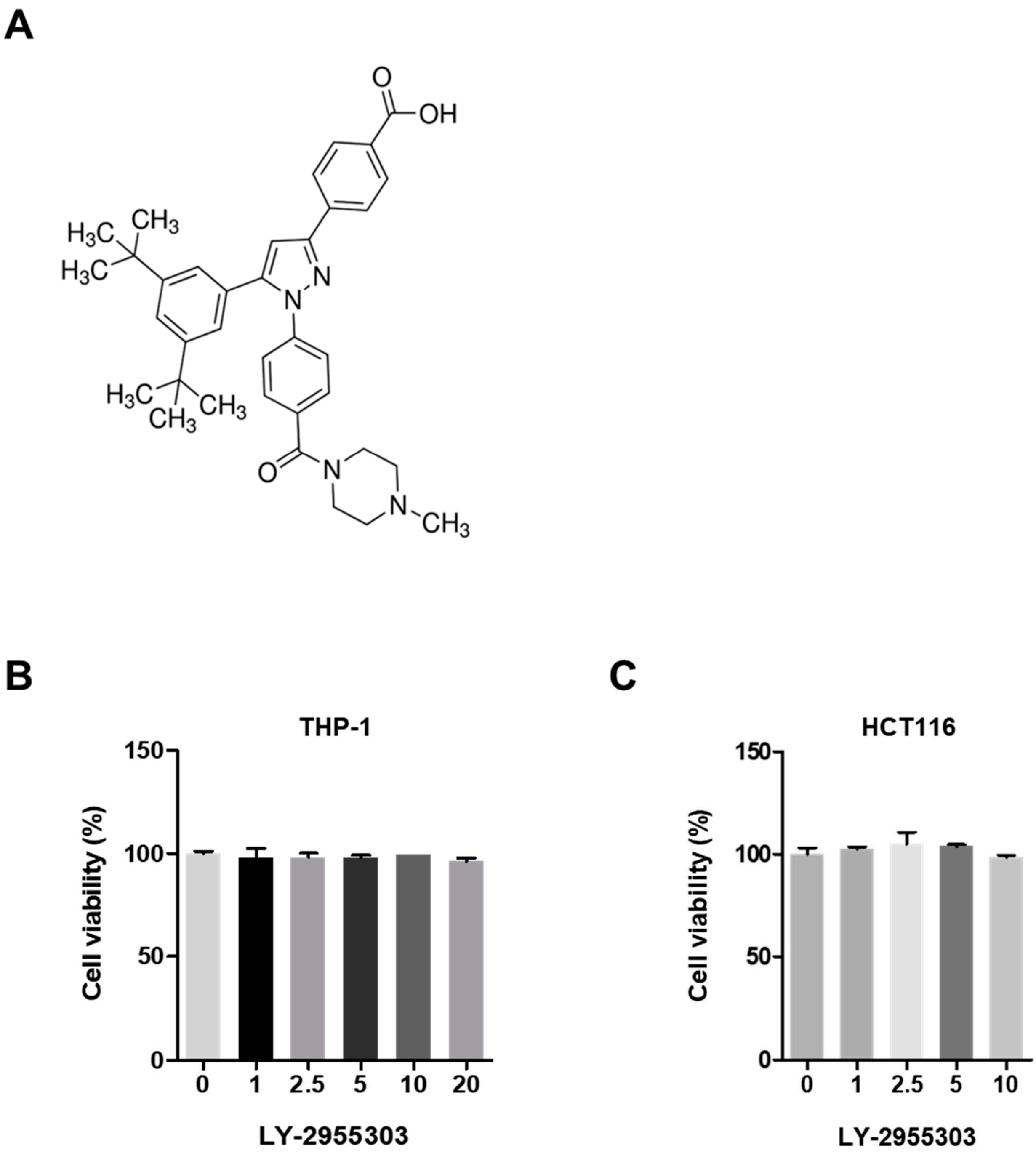

Suppl. Figure S3

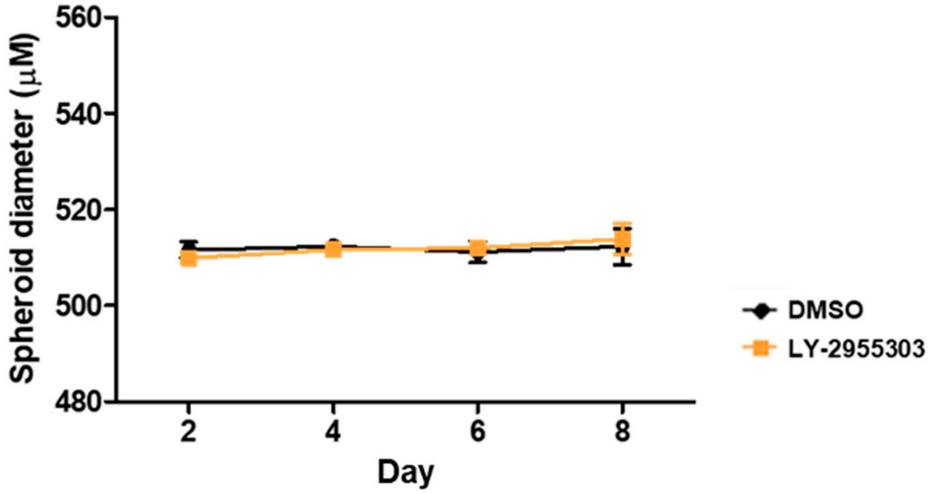

Supplementary Table

Supplementary Table S1. Relative mRNA expression of 42 genes in M2 compared to M0 THP-1 cells

Gene	Relative mRNA expression in M2 normalized to M0
THRA	3.0
RARA	1.9
RARB	2.3
RARG	1.7
PPARA	2.3
PPARD	2.3
PPARG	1.4
NR1D1	1.4
NR1D2	0.4
RORA	6.9
RORB	1.3
NR1F3	0.2
NR1H2	0.8
NR1H3	1.4
NR1H4	5.2
VDR	1.6
NR1I2	0.4
HNF4A	10.2
HNF4G	0.5
RXRA	1.4
RXRB	1.7
RXRG	20.4
NR2C1	0.6
NR2C2	0.7
NR2E1	0.8
NR2E3	1.8
NR2F1	0.9
NR2F2	4.1
ESR1	0.2
ESR2	2.5
ESRRA	1.5
ESRRB	4.8
ESRRG	0.3
NR3C1	0.5
AR	0.4
NR4A1	1.3
NR4A2	1.3
NR5A1	2.1
NR5A2	0.2

NR6A1	1.2
NR0B1	2.2
NR0B2	0.4
